# Impact of Fermentation, Autoclaving and Phytase Treatment on the Antioxidant Properties and Quality of Teff Cookies

**DOI:** 10.17113/ftb.61.03.23.8145

**Published:** 2023-09

**Authors:** İrem Karaçoban, Nermin Bilgiçli, Elif Yaver

**Affiliations:** 1 Department of Food Engineering, Engineering Faculty, Necmettin Erbakan University, Koycegiz Campus, Demeç Street, 42090 Konya, Turkey; 2 Department of Food Processing, Vocational School of Technical Sciences, Konya Technical University, İsmet Paşa Street, 42250 Konya, Turkey

**Keywords:** autoclaving, cookies, fermentation, phytase enzyme, phytic acid, teff [*Eragrostis tef* (Zucc.) Trotter]

## Abstract

**Research background:**

Teff [*Eragrostis tef* (Zucc.) Trotter] is an underutilised cereal crop grown mainly in Ethiopia and Eritrea. It is an excellent source of dietary fibre, vitamins, minerals and bioactive compounds. However, it also contains a high amount of phytic acid, which is an antinutrient and reduces the bioavailability of minerals and proteins. To improve the nutritional quality of teff, the phytic acid content should be reduced by an effective dephytinisation method.

**Experimental approach:**

In this study, various dephytinisation methods (fermentation, autoclaving and phytase treatment) were used to dephytinise teff flour. Undephytinised and dephytinised teff flour was mixed into wheat flour (0−40 %) to improve the functional properties of cookies. Twenty different cookie formulations were prepared according to 4x5x2 factorial design. The physical, chemical, nutritional and sensory properties of the cookies were investigated.

**Results and conclusions:**

Among the dephytinisation methods, fermentation produced the most effective reduction in phytic acid mass fraction (181 mg/100 g), followed by phytase treatment (198 mg/100 g). The protein, fat, Fe and Zn content and antioxidant activity of cookies enriched with dephytinised teff flour were comparable to cookies fortified with undephytinised teff flour. Moreover, the dephytinised teff cookies had lower phytic acid mass fractions. The cookies containing 40 % teff flour had higher antioxidant activity and nutritional quality than the control wheat cookies. The use of dephytinised teff flour reduced the spread ratio and the a* and b* values of cookies compared to undephytinised flour. Cookies containing fermented and phytase-treated teff flour had a harder texture than cookies containing undephytinised flour. In addition, as the amount of teff flour increased, the spread ratio values of cookies gradually incrased while their hardness decreased. Overall acceptability scores of cookies containing 10–20 % teff flour were similar to the control.

**Novelty and scientific contribution:**

To the best of our knowledge, this is the first study to determine the quality of cookies containing dephytinised teff flour. The data highlight the potential of dephytinised (especially autoclaved and phytase-treated) teff flour (up to 20 %) as a functional ingredient to enrich the mineral content and antioxidant capacity of foods. Furthermore, this study shows that fermentation, autoclaving and phytase treatment can be used to improve the nutritional quality of grains.

## INTRODUCTION

Teff [*Eragrostis tef* (Zucc.) Trotter] is an ancient gluten-free cereal grain. Ethiopia and Eritrea are the main producers of teff. In the 2020/21 production year, approx. 5510 kt of teff were produced in Ethiopia (*1*).It is also cultivated in India, Australia, USA, Canada and South Africa (*2*). Teff is rich in essential amino acids, unsaturated fatty acids, dietary fibre, vitamins, minerals (Ca, Fe, Mg and Zn) and phytochemicals (*3*, *4*). Teff grain has great potential to prevent various diseases such as malaria, anaemia and diabetes (*2*). Due to its health benefits and nutritional profile, teff has become increasingly important in recent years. It is a good option for the prepation of functional baked and extruded foods (*5*, *6*). Ziec *et al.* (*7*) found that the addition of 5, 10 and 15 % teff flour increased the concentrations of protein, dietary fibre, Fe, Ca, Mg and Mn in bread samples. Hager *et al.* (*8*) found that the use of teff flour improved the amounts of protein, ash and dietary fibre in gluten-free pasta compared to wheat pasta.

Despite the health benefits, consumption of teff is limited because of its antinutritional compounds, including phytic acid (*5*). Phytic acid [myo-inositol (1,2,3,4,5,6)-hexakisphosphate] is the major phosphate storage compound in cereals, legumes, oilseeds and nuts. It binds to minerals and proteins, reducing their bioavailability and digestibility (*9*). Therefore, dephytinisation methods such as fermentation, autoclaving and phytase treatment can be used to improve the bioavailability of nutrients in teff (*5*, *6*). Fermentation is a simple method to improve the functional and nutritional properties of foods by increasing the amount of free amino acids, available vitamins and minerals, and reducing the amount of anti-nutritional compounds (*10* ). Autoclaving is an inexpensive, simple and environmentally friendly technique for reducing the concentration of phytic acid in food. Özkaya *et al.* (*11*) reported a reduction of the phytic acid content in oat bran by about 95 % by autoclaving for 90 min. Exogenous phytase treatment is another effective method of decreasing the phytic acid content. Garcia-Mantrana *et al.* (*12*) found that the addition of the phytase caused a high degradation (approx. 91 %) of the phytic acid in bread.

Cookies are popular cereal-based products because of their availability, variety, practicability, low cost and long shelf life (*13*). However, the high fat and sugar content of cookies reduces their nutritional quality. With increasing public awareness, the demand for healthy and nutritionally enriched cookies has increased (*14*). In this context, the addition of functional ingredients to improve the nutritional quality of cookies has been extensively studied. Da Silva *et al.* (*13*) found that *Spirulina maxima* biomass can be used as a protein- and iron-rich ingredient in cookie formulations without affecting sensory acceptability. Giuffre *et al.* (*15*) reported that olive oil can effectively replace shortening to improve hardness, acidity, water activity values, unsaturated fatty acid content, antioxidant activity and sensory quality of cookies. Csutoras *et al.* (*16*) investigated the addition of lupin flour, which is a good source of protein and dietary fibre, to enriched gluten-free cookies. In another study, Pinto *et al.* (*17*) showed the potential of chestnut shell extract to produce acceptable functional cookies enriched with phenols. Lagana *et al.* (*18*) prepared functional cookies with bergamot by-products and found that the fortified cookies have stronger antioxidant capacity than the control.

Teff flour has great potential for preparing functional cookies due to its unique nutritional properties. Several authors have used it as an ingredient in cookies (*19*, *20*). Coleman *et al.* (*19*) studied the effect of using teff flour (10-100 %) on the fracture strength, spread factor and colour values of cookies. Joung *et al.* (*20*) reported colour, texture, sensory and antioxidant properties of cookies with 25, 50, 75 and 100 % teff flour. However, there are no studies on the addition of dephytinised brown teff flour in cookies.

To the best of our knowledge, this is the first study to compare the physical, textural, chemical and sensory quality and antioxidant properties of cookies made from dephytinised and undephytinised teff flour. The objectives of this study are to dephytinise teff flour by fermentation, autoclaving and phytase treatment, to develop nutritious cookie formulations with the addition of 0, 10, 20, 30 and 40 % dephytinised and undephytinised teff flour, and to determine the effects of dephytinisation methods and the amount of teff flour on the phytic acid content, antioxidant properties, chemical, physical, textural and sensory attributes of cookies.

## MATERIALS AND METHODS

### Materials

Brown teff seeds [*Eragrostis tef* (Zucc.) Trotter; Duru, Karaman, Turkey], icing sugar (Kenton, Istanbul, Turkey), baking powder (tetrasodium diphosphate and sodium hydrogen carbonate; Dr. Oetker, İzmir, Turkey), salt (Cihan, Konya, Turkey), skimmed milk powder (Enka, Konya, Turkey), vanilla (Dr. Oetker) and baker’s yeast (*Saccharomyces cerevisiae;* Pakmaya, Kocaeli, Turkey) were purchased from local shops in Konya, Turkey. Wheat flour (*Triticum compactum* Host.; Ova, Konya, Turkey) containing 0.68 % ash, 10.14 % protein, 1.08 % fat and 203 mg/100 g phytic acid and shortening (IFFCO, İzmir, Turkey) were obtained from a cookie factory in Karaman, Turkey. The phytase (*N*(FYT)=5000/g) was purchased from Novozymes (Bagsværd, Denmark).

### Dephytinisation of teff seeds

Teff seeds were ground into wholemeal flour using a laboratory mill (Arçelik-K3104; Istanbul, Turkey), divided into four parts and three parts of it were dephytinised by fermentation, autoclaving or phytase treatment methods. The untreated seed flour was called undephytinised teff flour.

#### Fermentation

Teff flour was mixed with distilled water at *m*(teff flour):*V*(water)=1:15. The slurry was mixed with 6 % yeast and fermented for 8 h at 30 °C in a water bath (Daihan Wisebath-WSB30; Gangwon, South Korea) (*21*).

#### Autoclaving

Teff flour and distilled water (1:3) were mixed. The pH of the slurry was adjusted to 4.5 with acetic acid (Sigma-Aldrich, Merck, Steinheim, Germany). Then the slurry was autoclaved (Daihan WiseClave Wac-60) at 121 °C for 60 min (*11*).

#### Phytase treatment

Teff flour (100 g) was mixed with 100 mL of 0.1 M acetic acid/sodium hydroxide buffer (pH=5.5; Sigma-Aldrich, Merck) and 0.5 g phytase. The slurry was made up to 1000 mL with distilled water (pH=5.5). The slurry was shaken in a water bath (Daihan Wisebath-WSB30) at 37 °C for 2 h (*22*).

At the end of dephytinisation, the slurries were filtered and dried (Nüve-KD200; Ankara, Turkey) overnight at 50 °C. All dried samples were ground with the laboratory mill (Arçelik-K3104).

### Preparation of cookies

The cookies were prepared according to AACC method 10-54 with some modifications (*23*). Cookies with 0 % teff flour (control) were made from 200 g wheat flour, 90 g icing sugar, 80 g shortening, 4 g baking powder, 2.5 g salt, 2 g skimmed milk powder, 1 g vanilla and water. The cookies enriched with teff flour were made by replacing wheat flour with 10, 20, 30 and 40 % undephytinised, fermented, autoclaved or phytase-treated teff flour separately.

All ingredients were mixed in a kneading machine (Hobart-N50; Ontario, Canada) for 5 min at low speed (speed 1) until a homogeneous dough was formed. The dough was stretched to a height of 5 mm, cut into a round shape with a diameter of 5 cm and baked (Vestel SF8401; Manisa, Turkey) at 175 °C for 15 min.

### Determination of phytic acid content

The phytic acid in the samples was extracted with 0.2 M hydrochloric acid (Merck, Darmstadt, Germany). The supernatant (0.5 mL) was reacted with 0.4 mM ammonium iron(III) sulfate (1 mL; Merck) and kept in boiling water bath for 30 min. Subsequently, the samples were incubated in an ice bath for 15 min. Then, 2 mL of 2,2’-bipyridine reagent (Merck) were added and the absorbance was measured at 519 nm (Biochrom Libra-S22; Cambridge, UK) (*24*).

### Determination of antioxidant properties

For extraction, the samples (2 g) were reacted with 20 mL of acidified methanol (*V*(methanol):*V*(HCl):*V*(distilled water)=80:1:10*;* Merck) in a water bath (Daihan Wisebath-WSB30) at room temperature ((25±2) °C) for 2 h. The extracts were centrifuged (1008×*g* for 10 min; Awel-MF20; Blain, France) (*25*).

For determination of total phenol content, the supernatant (0.1 mL) was mixed with 0.5 mL *φ*(Folin-Ciocalteu reagent)=10 % (Merck), 1.5 mL sodium carbonate solution (20 %; Merck) and 7.9 mL distilled water in a tube. The tube was kept at room temperature in the dark for 2.5 h. The absorbance values at 760 nm were expressed in mg gallic acid equivalents (GAE) per 100 g sample on dry mass basis using the calibration curve for gallic acid (Merck) (*26*).

For determination of antioxidant activity, the extract (0.1 mL) was reacted with 0.9 mL of 0.05 M Tris-HCl buffer (pH=7.4; Merck) and 2 mL of 0.1 mM DPPH solution (Merck). The blank was prepared with Tris-HCl+DPPH. The samples were stored at room temperature for 30 min and the absorbance (*A*) was measured (Biochrom Libra-S22) at 517 nm. The percentage of inhibition was determined using the following equation (*27*):

Antioxidant activity=[(*A*_blank_–*A*_sample_)/*A*_blank_]·100 /1/

### Chemical properties

Total ash mass fraction was measured in a muffle furnace (Daihan Wisetherm F12) at 550 °C according to AACC method 08-01 (*23*). Crude protein mass fraction was determined by the Dumas combustion method using Leco FP828 (St. Joseph, MI, USA) according to AACC method 46-30 (*23*). The crude fat mass fraction was determined according to AACC method 30-25 (*23*).

For mineral analysis, samples were mineralised with sulphuric and nitric acid solution (Merck) in a microwave oven (Mars 5; CEM Corporation, Matthews, NC, USA). The mass fractions of Ca, Fe, K, Mg, P and Zn were determined by ICP-AES (Varian Vista AX, Zug, Switzerland) according to Skujins (*28*).

Moisture content was determined according to AACC method 44-19 (*23*).

### Physical and textural properties

The diameter and thickness values were determined with a calliper (Mitutoyo, Tokyo, Japan) according to AACC method 10-54 (*23*). Spread ratio was calculated by dividing the diameter value by the thickness value of the cookies.

Cookie hardness was measured using a TA-XT.Plus texture analyser (Stable Micro Systems, Surrey, UK) coupled with a three-point bending rig (HDP/3PB) (*23*, *29*). The pre-test speed, test speed and post-test speed were set to 1.0, 3.0 and 10.0 mm/s, respectively. The test was carried out on at least five samples.

### Colour properties

Colour L* (lightness), a* (greenness/redness) and b* (blueness/yellowness) values were measured with a Minolta Chroma meter (CR-400; Osaka, Japan). Chroma (C*) values were calculated from:

C*=(a*^2^+b*^2^)^1/2^ /2/

Whiteness index (WI) was calculated using the following equation (*30*):

WI=100-[(100-L*)^2^+a*^2^+b*^2^]^1/2^ /3/

The determinations were made in five different positions on at least five samples.

### Sensory analysis

Sensory analysis was carried out by 12 experienced panellists (23-52 years old) from among the staff of the Food Engineering Department of Necmettin Erbakan University. For the evaluation, the samples were placed in a plastic dish coded with random digits. The colour, taste, odour, appearance and overall acceptability parameters of the cookies were rated on a 7-point scale (1=dislike very much, 7=like very much). Drinking water was provided to clean the palate (*31*).

### Statistical analysis

The teff flour and cookie data were compared by one-way and two-way analysis of variance (ANOVA), respectively, using Duncan’s multiple comparison test. The values of p<0.05 were accepted as significantly different. Data were the mean of triplicate determinations from duplicate experiments and were presented as mean±standard deviation.

## RESULTS AND DISCUSSION

### Phytic acid mass fraction, chemical properties and colour values of teff flour

The phytic acid mass fraction of dephytinised and undephytinised teff flour varied between 181 and 1350 mg/100 g (Table 1). Maximum reduction (86 %) in the phytic acid mass fraction of teff flour was achieved by fermentation, followed by phytase treatment (85 %) and autoclaving (69 %). The reduction in phytic acid mass fraction of fermented teff flour was possibly due to the action of the endogenous phytase enzyme in teff flour and the phytase activity of yeast (*12*). The reduction in the autoclaved sample could be due to the endogenous phytase activity of the teff flour and an increase in the solubility of the phytate complexes at high temperature and pressure and low pH in the autoclaving process (*4*). Özkaya *et al.* (*32*) found that the phytic acid content of rice bran decreased by about 93.9 % by autoclaving at 121 °C and pH=4.5 for 60 min. The efficiency of adding phytase in reducing phytic acid content was also reported by Rosa-Sibakov *et al.* (*33*), who found a decrease of over 80 % in the phytic acid content of faba bean by exogenous phytase (activity 20 U) treatment (55 °C, 1 h).

**Table 1:** Phytic acid mass fraction, antioxidant properties, macronutrients and colour values of undephytinised and dephytinised teff flour

Parameter	Undephytinised teff flour	Fermented teff flour	Autoclaved teff flour	Phytase-treated teff flour
*w*(phytic acid)/(mg/100 g)	(1350±6)^a^	(181±1)^d^	(419±3)^b^	(198±1)^c^
*w*(TPC as GAE)/(mg/100 g)	(547±7)^a^	(292±4)^b^	(256±3)^c^	(237±2)^d^
AA/%	(60.6±1.3)^a^	(40.2±2.0)^b^	(37.5±1.6)^b^	(37.1±1.5)^b^
*w*(ash)/%	(1.99±0.01)^a^	(1.72±0.04)^b^	(1.96±0.03)^a^	(1.89±0.03)^a^
*w*(protein)/%	(11.5±0.1)^a^	(11.6±0.1)^a^	(11.3±0.1)^a^	(10.9±0.1)^b^
*w*(fat)/%	(2.1±0.1)^a^	(2.0±0.1)^a^	(2.0±0.1)^a^	(2.1±0.1)^a^
L*	(63.71±0.07)^c^	(66.57±0.10)^b^	(56.75±0.06)^d^	(71.77±0.07)^a^
a*	(5.85±0.03)^b^	(4.45±0.02)^c^	(6.92±0.03)^a^	(3.40±0.05)^d^
b*	(13.33±0.05)^b^	(10.04±0.08)^c^	(14.17±0.06)^a^	(9.00±0.07)^d^
C*	(14.56±0.08)^b^	(10.98±0.05)^c^	(15.77±0.07)^a^	(9.62±0.06)^d^
WI	(60.90±0.07)^c^	(64.81±0.08)^b^	(53.96±0.06)^d^	(70.18±0.04)^a^

Mean values followed by different letters in superscript within a row are significantly (p<0.05) different. The results (except for colour) are based on dry mass. TPC=total phenol content, GAE=gallic acid equivalents, AA=antioxidant activity, C*=chroma, WI=whiteness index

All dephytinisation methods caused a decrease in total phenol content and antioxidant activity of teff flour (Table 1). This decrease could be due to the leaching of phenols into the water during the soaking and filtering steps of dephytinisation (*21*). Özkaya *et al.* (*11*) reported similar decreases in phenol content of dephytinised oat bran samples by fermentation and autoclaving. However, the antioxidant activities of dephytinised teff flour samples were statistically similar (p>0.05) (Table 1). A previous study on dephytinisation of cereal bran showed that autoclaving did not noticeably change the antioxidant activity of the samples compared to the control (*32*).

The ash mass fraction of teff flour ranged from 1.72 to 1.99 % (Table 1). Autoclaving and phytase treatments did not have a significant (p>0.05) effect on the ash mass fraction of teff flour. However, fermentation reduced the ash mass fraction of teff flour from 1.99 to 1.72 %. This result could be related to the leaching of soluble components during the soaking and filtering phases of the dephytinisation methods or the metabolic activities of the yeasts (*11*). The protein mass fraction of fermented (11.6 %) and autoclaved (11.3 %) teff flour was similar to that of undephytinised teff flour (11.5 %) (Table 1). However, the phytase treatment resulted in a reduction in the protein mass fraction of teff flour, probably due to the loss of dry matter during dephytinisation (*11*). The fat mass fractions of dephytinised teff flour were similar to those of undephytinised teff flour (Table 1).

It is observable in Table 1 that phytase-treated teff flour had the highest L* value and the lowest a* and b* values. These observations could be related to the chemical profile of the phytase-treated sample and the higher loss of pigments in the teff during the treatment with phytase (*34*). On the other hand, the lowest L* and the highest a* and b* values were observed in autoclaved teff flour (Table 1). Rico *et al.* (*35*) found similar results and observed a decrease in L* value and an increase in b* value of wheat bran after autoclaving. They explained these colour changes by the appearance of Maillard reaction products during autoclaving. The C* and WI values of the teff flour samples ranged from 9.62 to 15.77 and from 53.96 to 70.18, respectively (Table 1). The C* values of fermented and phytase-treated teff flour were lower than those of the undephytinised flour. Moreover, the WI value of undephytinised teff flour (60.90) increased after fermentation (64.81) and phytase treatment (70.18). The increase in WI value could be due to the loss of colour pigments in brown teff flour during dephytinisation (*34*). Surfiana *et al.* (*36*) also found an increase in WI value when fermenting cassava flour. On the other hand, autoclaving resulted in the highest C* and the lowest WI values in teff flour (Table 1), probably due to the formation of the Maillard reaction products during autoclaving (*35*). Similar to this study, Espinosa-Solis *et al.* (*37*) observed that autoclaving significantly increased the C* value of malanga flour, possibly due to the development of non-enzymatic browning reactions.

### Phytic acid content of cookies

Table S1 shows the phytic acid mass fractions of the cookies and Table 2 shows the effects of dephytinisation and teff flour level factors on the phytic acid content of cookies. The phytic acid mass fractions ranged from 69.0 to 413.8 mg/100 g (Table S1). All dephytinisation methods were effective in reducing the phytic acid mass fraction of cookies (Table 2). Compared to cookies made from undephytinised teff flour, the use of fermented and phytase-treated teff flour in cookies resulted in about 70 % lower phytic acid content. In addition, the use of autoclaved teff flour reduced the phytic acid mass fraction of cookies by 53 % compared to undephytinised sample. Baumgartner *et al.* (*21*) also reported a significant decrease in phytic acid content when 21 % dephytinised (fermented and autoclaved) oat bran samples were added to cookies.

**Table 2:** Effects of dephytinisation method and teff flour mass fraction on the phytic acid content and antioxidant properties of cookies

Factor	*N*	*w*(phytic acid)/(mg/100 g)	*w*(TPC as GAE)/ (mg/100 g)	AA/%
Dephytinisation method				
Undephytinised	10	(245±128)^a^	(128±32)^a^	(30.6±3.8)^a^
Fermented	10	(72±4)^c^	(108±20)^b^	(29.5±2.7)^a^
Autoclaved	10	(114±30)^b^	(112±17)^b^	(30.2±2.8)^a^
Phytase-treated	10	(72±3)^c^	(107±17)^b^	(30.1±2.4)^a^
*w*(teff flour)/%				
0 (control)	8	(73±2)^e^	(89±3)^e^	(26.4±1.4)^c^
10	8	(99±37)^d^	(98±6)^d^	(28.6±1.8)^bc^
20	8	(128±77)^c^	(112±13)^c^	(30.5±1.4)^ab^
30	8	(154±115)^b^	(127±16)^b^	(31.8±1.5)^a^
40	8	(176±151)^a^	(144±17)^a^	(33.3±2.1)^a^

Mean values followed by different letters in superscript within a column are significantly (p<0.05) different. Duncan’s multiple comparison test according to two-way analysis of variance. Values are the mean of triplicate determinations obtained from duplicate experiments. The results are based on dry mass. *N*=number of samples analysed, TPC=total phenol content, GAE=gallic acid equivalents, AA=antioxidant activity

As for the factor teff flour mass fraction, as the mass fraction of teff flour increased, the average phytic acid mass fraction of the cookies gradually increased (Table 2), mainly due to the high phytic acid content of undephytinised teff flour (Table 1). Köten (*38*) found a similar trend for the phytic acid content of tarhana enriched with 0, 20, 40, 60, 80 and 100 % teff flour.

### Antioxidant properties of cookies

Total phenol content and antioxidant activity of cookies are summarised in Table S1 and Table 2. With regard to dephytinisation method, the addition of dephytinised teff flour reduced the mean total phenol content of the cookies compared to undephytinised teff flour (Table 2). This is possibly due to the lower phenol content of dephytinised teff flour than undephytinised teff flour (Table 1). However, dephytinisation methods did not show any adverse effects on the antioxidant activity of the cookies (Table 2). Baumgartner *et al.* (*21*) reported similar observations for phenol content and antioxidant activity after replacing undephytinised oat bran with fermented and autoclaved bran in cookies.

As shown in Table 2, the mean total phenol content of the cookies increased with the increase in the amount of teff flour. This was probably due to the fact that teff flour contains a large amount of phenols. The antioxidant activity of cookies containing 10 % teff flour was close to the control (0 % teff flour) (Table 2). Furthermore, replacing wheat flour with higher mass fractions of teff flour (20-40 %) in cookies resulted in stronger antioxidant activity than the control. The result could be attributed to the high antioxidant activity of teff flour (Table 1). Homem *et al.* (*4*) found that bread made from 100 % teff flour had a higher antioxidant capacity and phenol content than bread made from 100 % wheat flour.

### Chemical properties of cookies

Ash, protein and fat contents of the cookies are given in Table S2. The effects of dephytinisation method and teff flour amount on ash, protein and fat mass fractions are shown in Table 3. Regarding the dephytinisation method, the addition of autoclaved teff flour resulted in a similar ash mass fraction in the cookies as in the samples made from undephytinised teff flour (Table 3). However, the use of fermented and phytase-treated teff flour resulted in a slight reduction in ash mass fraction in the cookies compared to the cookies with undephytinised flour. The results could be due to a slightly lower ash mass fraction of fermented and phytase-treated teff flour than that of undephytinised flour (Table 1). On the other hand, there were no differences in protein and fat mass fraction between the cookies made from undephytinised and dephytinised teff flour (Table 3).

**Table 3:** Effects of dephytinisation method and teff flour mass fraction on the nutrient composition of cookies

Factor	*N*	*w*(ash)/%	*w*(protein)/%	*w*(fat)/%
Dephytinisation method				
Undephytinised	10	(1.67±0.09)^a^	(5.64±0.14)^a^	(21.3±0.4)^a^
Fermented	10	(1.59±0.06)^c^	(5.70±0.12)^a^	(21.0±0.3)^a^
Autoclaved	10	(1.67±0.11)^a^	(5.66±0.10)^a^	(21.1±0.3)^a^
Phytase-treated	10	(1.62±0.09)^bc^	(5.62±0.09)^a^	(21.0±0.5)^a^
*w*(teff flour)/%				
0 (control)	8	(1.53±0.03)^c^	(5.57±0.09)^b^	(20.8±0.4)^c^
10	8	(1.58±0.04)^b^	(5.59±0.07)^b^	(20.9±0.3)^bc^
20	8	(1.63±0.05)^b^	(5.66±0.08)^ab^	(21.1±0.2)^abc^
30	8	(1.71±0.07)^a^	(5.71±0.10)^ab^	(21.3±0.3)^ab^
40	8	(1.75±0.06)^a^	(5.76±0.12)^a^	(21.5±0.3)^a^

Mean values followed by different letters in superscript within a column are significantly (p<0.05) different. Duncan’s multiple comparison test according to two-way analysis of variance. Values are the mean of triplicate determinations obtained from duplicate experiments. The results are based on dry mass. *N*=number of samples analysed

Regarding the teff flour level, the addition of 40 % teff flour increased the ash, protein and fat content of the cookies compared to the control (Table 3). These results could be due to the higher ash, protein and fat mass fraction of teff flour (Table 1) than of wheat flour (0.68 % ash, 10.14 % protein and 1.08 % fat). Thus, the data show that replacing wheat flour with teff flour improves the nutritional profile of the cookies. Our results agree with those of Köten (*38*), who found an increase in ash, protein and fat content after the addition of 40 % teff flour in tarhana.

### Mineral composition of cookies

Mineral composition (Ca, Fe, K, Mg, P and Zn) of cookies and effects of dephytinisation methods and teff flour levels on the mineral mass fraction are shown in Table S3 and Table 4, respectively. While cookies made from fermented and autoclaved teff flour had a slightly higher Ca mass fraction than cookies made from undephytinised flour, cookies with phytase-treated teff flour had the highest Fe mass fraction (Table 4). The degradation of phytates during phytase treatment could improve Fe bioaccessibility (*39*). On the other hand, Liang *et al.* (*40*) found that the increase in mineral content was due to a proportional increase because of the loss of soluble compounds during soaking. Compared to cookies made from undephytinised teff flour, cookies made from dephytinised teff flour had lower K and P values (Table 4). The decrease in mineral content could be because of the leakage of soluble solids during soaking and filtering treatments in dephytinisation processes (*11*). The dephytinisation methods did not show a significant (p>0.05) effect on the Zn content of cookies (Table 4).

**Table 4:** Effects of dephytinisation method and teff flour mass fraction on mineral composition of cookies

Factor	*N*	*w*(mineral)/(mg/100 g)					
Ca	Fe	K	Mg	P	Zn
Dephytinisation method							
Undephytinised	10	(43.9±5.8)^b^	(1.7±0.4)^b^	(131.8±22.8)^a^	(38.7±9.3)^a^	(272±30)^a^	(0.6±0.2)^a^
Fermented	10	(44.4±5.1)^a^	(1.8±0.4)^b^	(112.0±7.1)^c^	(32.1±3.5)^c^	(248±12)^d^	(0.6±0.2)^a^
Autoclaved	10	(44.7±6.0)^a^	(1.7±0.3)^b^	(120.6±16.8)^b^	(38.8±8.0)^a^	(268±29)^b^	(0.6±0.2)^a^
Phytase-treated	10	(42.2±4.0)^c^	(2.2±0.7)^a^	(111.3±8.2)^c^	(33.2±4.1)^b^	(252±16)^c^	(0.6±0.2)^a^
*w*(teff flour)/%							
0 (control)	8	(36.9±0.8)^e^	(1.3±0.1)^d^	(100.4±2.3)^e^	(27.6±0.7)^e^	(231±2)^e^	(0.4±0.1)^b^
10	8	(40.2±0.8)^d^	(1.5±0.1)^d^	(108.6±4.5)^d^	(31.0±2.0)^d^	(246±6)^d^	(0.5±0.1)^b^
20	8	(44.0±2.4)^c^	(1.8±0.2)^c^	(121.2±10.9)^c^	(36.0±3.5)^c^	(260±12)^c^	(0.6±0.1)^b^
30	8	(47.6±1.7)^b^	(2.1±0.3)^b^	(128.6±15.0)^b^	(40.8±5.2)^b^	(276±17)^b^	(0.8±0.1)^a^
40	8	(50.3±2.1)^a^	(2.4±0.5)^a^	(135.8±15.9)^a^	(43.1±6.8)^a^	(289±21)^a^	(0.8±0.1)^a^

Mean values followed by different letters in superscript within a column are significantly (p<0.05) different. Duncan’s multiple comparison test according to two-way analysis of variance. Values are the mean of triplicate determinations obtained from duplicate experiments. *N*=number of samples analysed. All values are expressed on dry mass basis

In general, increasing mass fractions of teff flour considerably increased the mass fraction of Ca, Fe, K, Mg and P of cookies (Table 4), showing the potential of teff flour to improve the mineral composition of cookies. Cookies containing 10 and 20 % teff flour had similar Zn mass fraction to the control, but the Zn mass fraction of cookies containing 40 % teff flour was almost twice that of the control. These results could be due to the richer mineral composition of teff flour than wheat flour (*6*). Ziec *et al.* (*7*) also reported increased Ca, K, Fe, P and Mg amounts in the production of bread in which 15 % of the wheat flour was replaced by teff flour.

### Moisture, physical and textural properties of cookies

The moisture content of cookies ranged between 2.58 and 4.71 % (Table S4). Cookies containing dephytinised teff flour had a slightly lower moisture content than cookies containing undephytinised teff flour (Table 5). Regarding teff flour amount, moisture content of cookies decreased with the increase of the mass fraction of teff flour. Coleman *et al.* (*19*) noted that teff flour does not have a great water absorption capacity.

**Table 5:** Effects of dephytinisation method and teff flour mass fraction on moisture content, physical properties, hardness and colour values of cookies

Factor	*N*	*w*(moisture)/%	*d*/mm	Thickness/mm	Spread ratio	Hardness/g	L*	a*	b*
Dephytinisation method									
Undephytinised	10	(3.8±0.6)^a^	(59.8±1.2)^a^	(5.9±0.5)^b^	(10.2±1.1)^a^	(2770±493)^c^	(58.4±9.5)^b^	(4.9±2.4)^a^	(18.8±3.4)^a^
Fermented	10	(3.6±0.3)^b^	(59.0±1.0)^b^	(6.2±0.5)^a^	(9.6±0.9)^b^	(3040±427)^a^	(57.4±10.4)^c^	(4.7±2.4)^b^	(17.2±3.9)^b^
Autoclaved	10	(3.2±0.5)^c^	(58.6±0.3)^d^	(6.0±0.4)^ab^	(9.7±0.6)^b^	(2531±568)^d^	(58.7±9.7)^b^	(4.6±2.3)^c^	(17.2±3.8)^b^
Phytase-treated	10	(3.2±0.6)^c^	(58.8±0.6)^c^	(6.2±0.3)^a^	(9.5±0.5)^b^	(3000±555)^b^	(59.5±9.4)^a^	(4.4±2.2)^d^	(16.7±4.1)^c^
*w*(teff flour)/%									
0 (control)	8	(4.0±1.1)^a^	(58.2±0.2)^e^	(6.6±0.1)^a^	(8.8±0.2)^e^	(3576±23)^a^	(74.2±0.1)^a^	(0.6±0.0)^e^	(23.9±0.1)^a^
10	8	(3.7±0.7)^b^	(58.5±0.4)^d^	(6.3±0.3)^a^	(9.2±0.4)^d^	(3074±421)^b^	(62.7±0.7)^b^	(4.1±0.3)^d^	(18.6±1.2)^b^
20	8	(3.4±0.2)^c^	(58.9±0.8)^c^	(6.0±0.3)^b^	(9.8±0.6)^c^	(2792±344)^c^	(56.2±1.6)^c^	(5.6±0.4)^c^	(16.2±1.4)^c^
30	8	(3.2±0.4)^d^	(59.5±0.6)^b^	(5.8±0.2)^bc^	(10.2±0.4)^b^	(2464±216)^d^	(51.3±1.1)^d^	(6.2±0.4)^b^	(14.8±1.1)^d^
40	8	(3.0±0.4)^e^	(60.0±1.0)^a^	(5.6±0.3)^c^	(10.7±0.7)^a^	(2269±183)^e^	(48.3±0.9)^e^	(6.6±0.2)^a^	(13.9±0.8)^e^

Mean values followed by different letters in superscript within a column are significantly (p<0.05) different. Duncan’s multiple comparison test according to two-way analysis of variance. Values are the mean of triplicate determinations obtained from duplicate experiments. *N*=number of samples analysed

The addition of dephytinised teff flour reduced the diameter and spread ratio of the cookies compared to the cookies with undephytinised flour (Table 5). On the other hand, thickness of cookies increased slightly when dephytinised teff flour (except for autoclaved flour) was used instead of undephytinised flour. The results can be attributed to alterations in the chemical composition of dephytinised teff flour because of the heat treatments, enzymatic reactions and also the leakage of soluble compounds, which can affect the dough viscosity, during soaking and filtering steps in dephytinisation methods (*41*).

In terms of the factor teff flour mass fraction, with the increase of teff flour mass fraction, the diameter and spread ratio of cookies increased gradually (Table 5). The cookies made with 10 % teff flour had a similar thickness to the control. However, the addition of higher mass fractions (20-40 %) of teff flour reduced the thickness of cookies. The results could be due to the reduction of the gluten content in the cookies by the addition of teff flour. Gluten in the cookies acts as a binding agent and increases the viscosity of the dough (*42*). The dilution of gluten, the disruption of the gluten network and the decrease in dough viscosity due to the addition of gluten-free teff flour could be responsible for the increase in diameter and spread ratio and the decrease in thickness of cookies with teff flour (*21*, *43*). The study by Coleman *et al.* (*19*) also found that spread ratio values of cookies increased with increasing teff flour content and suggested that this result was probably due to the low water absorption capacity of teff flour. In general, high spread ratio value in cookies is a desirable goal (*44*).

Texture analysis showed that the addition of fermented teff flour gave the hardest texture in the cookies (Table 5). On the other hand, cookies made with phytase-treated teff flour had a harder texture than the cookies made with undephytinised teff flour. The interactions between protein, starch and water have an impact on the hardness of cookies (*43*). The differences in the hardness values of cookies (Table 5) could be related to the changes in the composition of teff flour during dephytinisation.

The hardness of cookies gradually decreased as the mass fraction of teff flour increased, probably due to the reduction in gluten content caused by the addition of teff flour. The lower formation of the gluten network could lead to a lower hardness of cookies (*45*). Joung *et al.* (*20*) found similar results after replacing wheat flour with 25, 50, 75 and 100 % teff flour in the production of cookies.

### Colour values of cookies

The L*, a*, and b* values of the cookies ranged from 46.9–74.2, 0.59–6.86 and 13.05–23.96, respectively (Table S4). As shown in Table 5, cookies made with phytase-treated teff flour had the highest L* (59.5) and the lowest a* (4.4) values. Moreover, L* values of cookies enriched with autoclaved teff flour were similar to those of cookies enriched with undephytinised teff flour. On the other hand, the a* and b* values of the cookies decreased with the use of dephytinised flour compared to the cookies made with undephytinised teff flour. This decrease could be attributed to the leakage of soluble proteins and pigments during soaking and filtering steps in dephytinisation processes, which reduces the occurrence of Maillard reaction products in the cookies made with dephytinised teff flour (*34*). Oyeyinka *et al.* (*45*) reported that cookies made with unfermented cassava flour had higher a* and b* values than cookies made with fermented cassava flour. Similarly, Baumgartner *et al.* (*21*) found a decrease in the a* and b* values of cookies when untreated oat bran was replaced with fermented or autoclaved bran samples.

The addition of teff flour reduced the L* and b* values but increased the a* values of cookies (Table 5). The colour changes may be due to the natural colour of teff flour. Coleman *et al.* (*19*) observed similar trends in colour values when they investigated the impact of teff flour addition on the colour of cookies. Lu *et al.* (*14*) also came to a similar conclusion that cookies with chickpea flour had lower L* and b* values and higher a* values than the control. The authors pointed out that the higher protein content of chickpea flour trigger the Maillard reaction, resulting in a darker colour of cookies.

### Sensory properties of cookies

Due to their better technological properties, cookies containing 0 % teff flour (control), undephytinised teff flour (10–30 %), autoclaved teff flour (10–30 %) and teff flour treated with phytase (10–30 %) were subjected to sensory evaluation.

**Fig. 1:**
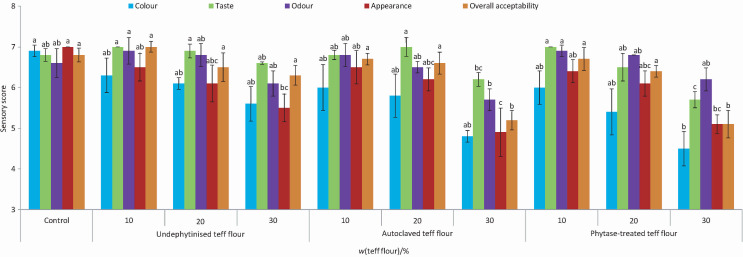
Sensory evaluation of cookies

The colour scores of the cookies made with all tested mass fractions of undephytinised and autoclaved teff flour were similar to the control (Fig. 1). The colour scores of cookies made with 10 and 20 % teff flour treated with phytase were close to those of the control, while the cookies made with 30 % of this flour had a lower colour score than the control. The taste and odour scores of cookies made with undephytinised, autoclaved (except for 30 %) and phytase-treated (except for 30 %) teff flour were close to those of the control. When up to 20 % teff flour was added to the cookies, the appearance scores were close to those of the control. However, the addition of 30 % teff flour reduced the appearance score of cookies compared to the control, probably due to the darker colour of the cookies with 30 % teff flour (Table 5). The addition of all tested mass fractions of undephytinised teff flour resulted in cookies with similar overall acceptability scores to the control (Fig. 1). Cookies made with 10 and 20 % autoclaved and phytase-treated teff flours did not show any negative impact on overall acceptability. However, the use of 30 % autoclaved and phytase-treated teff flour showed the lowest overall acceptability scores. Joung *et al.* (*20*) also found that the use of 25, 50, 75 and 100 % teff flour in cookies did not have an adverse effect on the overall acceptance score of cookies.

## CONCLUSIONS

The data obtained in this study indicate that dephytinised teff flour can be considered as a promising functional ingredient as it is rich in ash and phenols and low in phytic acid. Moreover, the replacement of undephytinised teff flour with dephytinised flour significantly reduced phytic acid content of cookies without adversely affecting protein, fat, Fe and Zn mass fractions. Among the dephytinisation methods, fermentation and phytase treatment resulted in the lowest phytic acid mass fraction in cookies made from teff flour. On the other hand, autoclaving resulted in higher ash mass fraction in the cookies made from teff flour than the other dephytinisation methods. The addition of teff flour to the cookie formulation showed a notable improvement in antioxidant capacity and mineral composition. Cookies made with teff flour also had acceptable physical properties. Cookies made with dephytinised teff flour had lower spread ratio values than cookies made with undephytinised flour. The cookies made with fermented flour had the hardest texture (3040 g), while autoclaving gave the softest texture (2531 g). The sensory evaluation showed that the use of undephytinised (up to 30 %), autoclaved (up to 20 %) and phytase-treated teff flour (up to 20 %) could allow the preparation of cookies with comparable sensory properties to the control. The results suggest that it is likely to be possible to produce healthy and nutritious cookies with acceptable technological and sensory quality using dephytinised (especially autoclaved and phytase-treated) teff flour (up to 20 %).

## Appendix

**Table S1:** Phytic acid content and antioxidant properties of cookies

Dephytinisation method	*w*(teff flour)/%	*w*(phytic acid)/(mg/100 g)	*w*(TPC as GAE)/ (mg/100 g)	AA/%
Undephytinised	0	72.3±1.8	88.7±1.0	26.1±2.0
	10	156.7±2.4	100.2±4.0	28.1±3.0
	20	249.1±5.8	129.1±5.8	31.3±1.9
	30	334.6±6.2	152.7±3.8	32.4±2.2
	40	413.8±5.9	169.4±11.9	35.3±2.5
Fermented	0	73.2±3.1	88.4±3.4	26.0±1.4
	10	72.6±3.7	92.4±4.8	27.9±0.1
	20	73.9±5.5	99.7±6.7	29.5±0.6
	30	70.1±4.1	121.7±2.4	31.4±2.2
	40	71.4±5.1	138.4±2.0	32.5±2.1
Autoclaved	0	71.9±1.6	88.0±5.7	26.3±1.9
	10	92.8±3.1	103.4±4.8	29.2±2.6
	20	118.0±4.2	114.4±6.2	30.6±0.3
	30	137.3±3.8	118.1±4.3	32.2±1.2
	40	151.7±4.7	134.6±3.5	32.8±1.7
Phytase-treated	0	73.0±2.8	89.0±5.6	27.0±1.4
	10	73.2±2.6	96.1±7.2	29.3±1.9
	20	71.9±4.4	103.4±4.8	30.4±2.3
	30	74.6±0.6	114.4±3.7	31.0±1.4
	40	69.0±5.7	132.7±3.9	32.6±2.2

Values are the average of triplicate measurements of the duplicate samples. The results are expressed on dry mass basis. TPC=total phenol content, GAE=gallic acid equivalents, AA=antioxidant activity

**Table S2:** Nutrient composition of cookies

Dephytinisation method	*w*(teff flour)/%	*w*(ash)/%	*w*(protein)/%	*w*(fat)/%
Undephytinised	0	1.55±0.03	5.6±0.2	20.8±0.1
	10	1.62±0.04	5.5±0.1	21.0±0.3
	20	1.67±0.03	5.6±0.1	21.2±0.3
	30	1.73±0.04	5.7±0.1	21.6±0.2
	40	1.79±0.01	5.8±0.2	21.8±0.2
Fermented	0	1.51±0.00	5.6±0.1	20.7±0.2
	10	1.55±0.06	5.6±0.1	20.9±0.3
	20	1.58±0.01	5.7±0.1	21.1±0.1
	30	1.63±0.04	5.8±0.1	21.2±0.2
	40	1.66±0.01	5.8±0.1	21.4±0.3
Autoclaved	0	1.53±0.03	5.6±0.1	20.8±0.2
	10	1.59±0.06	5.6±0.0	21.0±0.4
	20	1.65±0.07	5.7±0.1	21.2±0.2
	30	1.78±0.04	5.7±0.1	21.2±0.3
	40	1.79±0.01	5.7±0.1	21.3±0.4
Phytase-treated	0	1.52±0.04	5.6±0.1	20.8±1.1
	10	1.57±0.01	5.6±0.1	20.8±0.3
	20	1.61±0.06	5.6±0.0	21.0±0.2
	30	1.68±0.00	5.6±0.1	21.1±0.2
	40	1.74±0.01	5.7±0.1	21.5±0.2

Values are the average of triplicate measurements of the duplicate samples. The results are expressed on dry mass basis

**Table S3:** Mineral composition of cookies

Dephytinisation method	*w*(teff flour)/%	*w*(mineral)/(mg/100 g)					
Ca	Fe	K	Mg	P	Zn
Undephytinised	0	36.4±0.6	1.3±0.1	99.2±2.0	27.6±0.8	230.2±2.6	0.4±0.1
	10	40.8±1.1	1.4±0.0	115.3±1.8	30.5±0.7	250.4±1.6	0.5±0.1
	20	42.6±0.8	1.7±0.0	137.9±2.0	38.9±0.8	275.6±2.3	0.5±0.0
	30	47.2±0.3	2.0±0.1	149.6±0.8	45.6±0.8	294.8±2.6	0.7±0.1
	40	52.4±0.6	2.2±0.3	157.2±1.7	50.8±1.1	309.2±1.7	0.9±0.2
Fermented	0	37.6±0.8	1.2±0.1	103.2±1.0	28.3±0.4	232.4±3.4	0.4±0.1
	10	40.5±0.7	1.5±0.0	105.7±0.4	29.1±0.6	240.4±2.3	0.4±0.1
	20	45.6±0.8	1.9±0.1	113.6±1.6	31.4±0.6	245.1±1.6	0.5±0.1
	30	47.8±1.1	2.1±0.1	116.1±1.3	35.6±0.8	258.4±2.0	0.7±0.3
	40	50.7±1.0	2.2±0.1	121.3±1.0	36.3±0.4	265.6±2.3	0.8±0.1
Autoclaved	0	36.2±0.3	1.3±0.1	98.6±2.0	27.3±0.4	229.6±1.6	0.5±0.1
	10	40.3±0.4	1.5±0.1	108.3±1.8	33.9±0.6	251.7±0.4	0.5±0.2
	20	46.7±1.0	1.6±0.1	120.7±0.4	39.2±0.4	264.3±1.8	0.6±0.0
	30	49.6±0.8	1.9±0.1	132.9±1.6	45.6±0.8	288.5±2.1	0.8±0.1
	40	50.9±1.3	2.0±0.3	142.3±1.0	47.9±0.6	307.7±2.8	0.8±0.0
Phytase-treated	0	37.4±0.6	1.4±0.1	100.8±1.7	27.1±0.6	231.4±2.3	0.4±0.1
	10	39.2±0.3	1.6±0.0	105.1±1.6	30.5±0.7	240.6±2.0	0.5±0.0
	20	41.3±0.4	2.2±0.0	112.4±2.0	34.6±0.8	253.7±2.4	0.6±0.0
	30	45.6±0.8	2.5±0.1	115.7±1.0	36.5±0.7	261.7±1.8	0.8±0.1
	40	47.3±0.4	3.1±0.3	122.6±1.6	37.5±0.7	272.9±2.7	0.9±0.1

Values are the average of triplicate measurements of the duplicate samples

**Table S4:** Moisture content, physical properties, hardness and colour values of cookies

Dephytinisation method	*w*(teff flour)/%	*w*(moisture)/%	*d*/mm	Thickness/mm	Spread ratio	Hardness/g	*L**	*a**	*b**
Undephytinised	0	4.09±0.03	58.2±0.2	6.60±0.05	8.8±0.1	3571±14	74.2±0.2	0.61±0.01	23.92±0.07
	10	4.71±0.10	58.9±0.3	6.27±0.02	9.4±0.2	2910±8	62.1±0.2	4.51±0.04	20.51±0.11
	20	3.45±0.07	60.1±0.1	5.71±0.06	10.5±0.1	2585±5	55.4±0.2	6.22±0.03	18.41±0.06
	30	3.74±0.03	60.5±0.1	5.65±0.04	10.7±0.1	2622±22	51.7±0.1	6.54±0.01	16.25±0.13
	40	3.13±0.07	61.1±0.1	5.25±0.13	11.6±0.1	2158±18	48.8±0.1	6.86±0.05	14.83±0.03
Fermented	0	3.95±0.18	58.1±0.1	6.67±0.06	8.7±0.2	3565±5	74.0±0.1	0.62±0.01	23.87±0.02
	10	3.61±0.06	58.3±0.3	6.68±0.03	8.7±0.1	3360±20	62.5±0.2	3.86±0.06	18.24±0.01
	20	3.41±0.13	58.5±0.3	6.34±0.09	9.2±0.1	3116±4	54.1±0.4	5.76±0.06	16.02±0.07
	30	3.36±0.08	59.4±0.1	5.73±0.08	10.4±0.1	2655±10	49.7±0.1	6.60±0.03	15.04±0.08
	40	3.41±0.06	60.6±0.1	5.63±0.12	10.8±0.1	2502±13	46.9±0.1	6.45±0.01	13.05±0.08
Autoclaved	0	4.05±0.10	58.2±0.2	6.59±0.16	8.8±0.1	3559±6	74.3±0.0	0.60±0.00	23.84±0.02
	10	3.37±0.08	58.4±0.1	6.18±0.21	9.5±0.1	2511±8	62.4±0.1	4.20±0.03	17.99±0.21
	20	3.25±0.07	58.5±0.2	5.86±0.08	10.0±0.2	2374±6	57.2±0.2	5.28±0.07	15.65±0.10
	30	2.97±0.03	58.9±0.1	5.83±0.14	10.1±0.1	2148±11	51.3±0.1	6.05±0.04	13.85±0.07
	40	2.58±0.03	58.8±0.1	5.75±0.27	10.2±0.2	2062±13	48.3±0.2	6.65±0.05	14.53±0.08
Phytase-treated	0	4.10±0.08	58.2±0.2	6.62±0.07	8.8±0.1	3608±10	74.2±0.1	0.59±0.00	23.96±0.10
	10	3.09±0.11	58.3±0.3	6.23±0.36	9.4±0.3	3515±5	63.7±0.2	3.93±0.06	17.63±0.06
	20	3.59±0.08	58.5±0.1	6.20±0.19	9.4±0.1	3094±14	57.9±0.2	5.19±0.06	14.91±0.11
	30	2.67±0.03	59.3±0.1	6.05±0.07	9.8±0.1	2429±21	52.4±0.2	5.81±0.04	13.87±0.16
	40	2.78±0.16	59.5±0.1	5.95±0.04	10.0±0.2	2355±14	49.1±0.2	6.31±0.01	13.25±0.13

Values are the average of triplicate measurements of the duplicate samples
